# Distribution and Feeding of *Hexatilemonas jangsaensis*, a Novel Cosmopolitan Member of the Uncultured Marine Apusomonad Clade

**DOI:** 10.1111/1462-2920.70282

**Published:** 2026-03-19

**Authors:** Dong Hyuk Jeong, Hyeon Been Lee, Da Yeong Ji, Aaron A. Heiss, Jong Soo Park

**Affiliations:** ^1^ Department of Oceanography Kyungpook National University Daegu Republic of Korea; ^2^ Kyungpook Institute of Oceanography Kyungpook National University Daegu Republic of Korea

**Keywords:** apusomonads, feeding behaviour, global distribution, new genus, new species, taxonomy

## Abstract

The perplexing apusomonads, a sister lineage to Opisthokonta (including animals and fungi), are bacterivorous heterotrophic nanoflagellates whose diversity and ecological role remain poorly understood. Members of the large APU‐30 clade are found exclusively in marine environments and mostly comprise uncultured lineages. Here, we isolated a novel lineage within an uncultured subclade of APU‐30 from Korean coastal waters. Although the new isolate shares key morphological features with *Chelonemonas* in APU‐30, it possesses a differently segmented dorsal pellicle. Phylogenetic analyses placed this organism closest to the genetically distinct ‘*Thecamonas*’ sp. Bamfield. Based on a combination of morphological and genetic features, we propose a novel genus and species for this organism: *Hexatilemonas jangsaenesis* gen. et sp. n. This novel apusomonad captures bacteria with its lateral pseudopodia, showing a sit‐and‐wait feeding strategy, which probably provides an efficient way for utilising bacterial assemblages. Interestingly, environmental DNA surveys showed a widespread distribution of *Hexatilemonas*‐like sequences across global marine environments, occurring in 29.2% of epipelagic and 47.7% of mesopelagic samples, suggesting that this genus is cosmopolitan. Our findings expand the known diversity of apusomonads by describing a novel lineage and provide insights into previously uncharacterised lineages and their ecological roles in marine ecosystems.

## Graphical abstract

This study describes a novel species of marine heterotrophic nanoflagellate, *Hexatilemonas jangsaensis* gen. et sp. n., within the uncultured APU‐30 clade, and highlights the global distribution pattern of this previously uncharacterised genus. This isolate is hypothesised to feed on bacteria attached to substrates in the water column using pseudopodia. It shows higher relative abundance in epipelagic depths than in mesopelagic depths and represents a cosmopolitan genus.

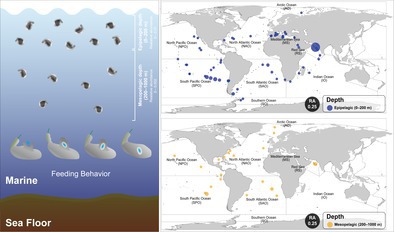

## 1 Introduction

Apusomonadida is an enigmatic lineage of heterotrophic nanoflagellates (2–20 μm in size) that bear two flagella and inhabit fresh water, moist soils, and marine environments, where they glide along substrates and ingest bacteria (Heiss et al. 2015; Torruella et al. 2017). Their first records, under light microscopy only, placed them in two morphotype‐defined genera: the fusiform *Amastigomonas* (Griessmann 1913) and the circular *Apusomonas* (Aléxéieff 1924). With the advent of scanning and transmission electron microscopy, ultrastructural features such as a semi‐rigid pellicle (undetectable by light microscopy) were revealed, and additional details of the cytoskeleton and flagellar apparatus were characterised (Vickerman et al. 1974; Karpov 2007; Heiss et al. 2013). Meanwhile, molecular phylogenetic analyses showed that the original morphological classification was oversimplified, with great genetic diversity contained within organisms assigned to *Amastigomonas* (Cavalier‐Smith and Chao 2010), a name no longer used in most treatments of Apusomonadida. As a result, the original two‐genus framework has been expanded to 11 genera: *Apusomonas*, *Cavaliersmithia*, *Catacumbia*, *Chelonemonas*, *Karpovia*, *Manchomonas*, *Multimonas*, *Mylnikovia*, *Podomonas*, *Singekia* and *Thecamonas* (Cavalier‐Smith and Chao 2010; Heiss et al. 2015; Torruella et al. 2023).

Apusomonadida is currently regarded as a sister lineage to Opisthokonta, the latter including animals, fungi, and their unicellular relatives (Brown et al. 2013; Torruella et al. 2025), a position that makes apusomonads pivotal for reconstructing the early morphological and metabolic evolution of eukaryotes. However, their taxonomic breadth and ecological roles remain underexplored because apusomonads occur at low abundance in environmental samples, and they are both small and easily confused with other, unrelated lineages, making them difficult to culture. Although only 29 species in 11 genera have been formally described in Apusomonadida, environmental 18S rRNA and metabarcoding surveys have recovered several unclassified apusomonad lineages (Torruella et al. 2017). Furthermore, their distribution in marine settings is still poorly constrained; for instance, thaumatomastigids together with apusomonads account for an interval as wide as 1%–20% of the heterotrophic flagellate biomass in benthic communities (Arndt et al. 2000). Since the 2000s, global circumnavigation programs such as the Malaspina‐2010 Expedition and Tara Oceans (2009–2013) have produced extensive metabarcoding datasets that now underpin large‐scale biogeographic studies of marine protists (Logares et al. 2020; Massana et al. 2021). Yet no study has systematically interrogated these databases to determine the worldwide distribution of Apusomonadida, nor has any work examined their feeding strategies or other ecological traits in a comprehensive manner.

In this study, we isolate a previously uncharacterised apusomonad from the large apusomonad APU‐30 clade collected in Korean coastal waters. We formally describe one new genus and one new species by: (1) analysing cell morphology and feeding behaviour with light microscopy and scanning electron microscopy (SEM), (2) reconstructing its phylogenetic position using 18S rRNA gene sequences, and (3) assessing its global distribution through metabarcoding data using the V4 region of 18S rDNA from the Tara Oceans database.

By integrating morphology, phylogenetics, and global metabarcoding, our work closes three long‐standing gaps in apusomonad research. First, the establishment of *Hexatilemonas jangsaenesis* gen. et sp. n. significantly expands the number of cultured lineages within the large APU‐30 clade, providing a living model for ultrastructural and genomic studies. Second, our phylogeny anchors a previously sequence‐only branch of Apusomonadida, clarifying the taxonomic framework needed for accurate annotation of environmental datasets. Third, the Tara Oceans survey reveals that *Hexatilemonas* is not a coastal rarity but a cosmopolitan player whose occurrence spans temperate and tropical water masses. Together, these advances suggest the ecological importance of apusomonads as bacterivores in benthic and planktonic food webs, supply new comparative material for testing early‐eukaryote evolutionary hypotheses, and lay groundwork for incorporating Apusomonadida into next‐generation biogeochemical and ecosystem models.

## 2 Materials and Methods

### 2.1 Sampling and Culturing

The apusomonad strain MHF056 was isolated on 16 June 2023 from surface‐water/sediment‐interface samples collected at Jangsa Beach, Yeongdeok, Korea (36°16′48.4″ N, 129°22′40.8″ E). A monoculture was established by manual single‐cell picking with a glass micropipette. The isolated cell was dispensed into a 48‐well culture plate (30,048, SPL Life Sciences Co. Ltd., Pocheon, Republic of Korea) containing medium prepared from coastal seawater taken at Yeongildae Beach in Korea. This seawater was filtered through glass‐fibre filters, autoclave‐sterilised, and supplemented with R2A (final concentration of 0.05%, MB‐R2230, Kisan Bio Co. Ltd., Seoul, Republic of Korea) during the single‐cell isolation. During subsequent subcultures, Guillard's Marine Water Enrichment Solution (F/2: final concentration 1×, G0154, Sigma‐Aldrich, St Louis, MO, United States) was added to promote prokaryotic growth. Cultures were incubated at 25°C under a 12 h light: 12 h dark photoperiod. After 7 days, the culture was transferred to flat 25‐mL plug‐cap cell culture flasks (70,175, SPL Life Sciences Co. Ltd., Pocheon, Republic of Korea) filled with the same medium with an added barley grain (to provide a bacterial food source) for batch propagation; subsequent subculturing was carried out every 2 weeks.

### 2.2 Microscopy

Samples of 20 μL of live‐cell suspension were pipetted onto glass slides, covered with a coverslip, and observed with differential interference contrast and phase‐contrast optics using a Leica DM5500B microscope equipped with a DFC550 digital camera (Leica, Wetzlar, Germany). Morphological features, including cell dimensions and flagellar length, were measured from the resulting digital images using ImageJ v2.9.0 (WS Rasband, US National Institutes of Health, Bethesda, MD, USA; https://imagej.net).

For scanning electron microscopy, 3 mL of culture was centrifuged at 2000 × *g*, and the supernatant fixed in 2.5% glutaraldehyde (v/v, electron microscopy grade, Sigma‐Aldrich, St Louis, MO, United States) for 2 h at 4°C. A 200 μL aliquot of the fixed suspension was allowed to settle onto glass coverslips coated with 0.1% poly‐L‐lysine (final concentration, P8920, Sigma‐Aldrich, St Louis, MO, United States) for 1 h, and then rinsed sequentially in artificial seawater (ASW), 50% ASW, and distilled water for 5 min each. For secondary fixation, osmium tetroxide (SPI Supplies, West Chester, PA, USA) was used at a 1% (w/v) final concentration on ice for 30 min, followed by three 5‐min washes in distilled water. Dehydration proceeded through an ethanol series of 10%, 30%, 50%, 70%, 90%, 95%, and 100% (three changes), with 5 min per step. Ethanol was then exchanged with tert‐butanol using an intermediate stage of 50% tert‐butanol and three changes of 100% tert‐butanol, again for 5 min each. Samples were dried overnight in a freeze‐dryer, sputter‐coated with platinum, and examined under an SU8220 field emission scanning electron microscope (Hitachi, Tokyo, Japan).

To examine bacterial feeding behaviour, a 0.5 mm‐thick silicone rubber sheet was punched to create a circular ring with an outer diameter of 10 mm and an inner diameter of 7 mm. The ring was fixed to a glass slide with Vaseline, and 25 μL of strain MHF056 was dispensed into its centre. After the preparation was sealed with a coverslip, feeding activity was observed under phase contrast with a Leica DM5500B microscope fitted with a DFC550 digital camera (Leica, Wetzlar, Germany). Video sequences were recorded using the screen‐capture function, and still frames and video clips were extracted and edited with Adobe Premiere Pro 2025 v25.2.3 (Adobe Inc., San Jose, CA).

### 2.3 DNA Extraction and PCR Amplification

Genomic DNA from strain MHF056 was extracted with the DNeasy Blood and Tissue Kit (Qiagen, Hilden, Germany) following the manufacturer's protocol. The 18S rDNA fragment was amplified with the primer pair EukA/EukB (Medlin et al. 1988). PCR was performed with Takara Ex Taq DNA polymerase (Takara, Shiga, Japan) under the following cycling regime: (1) 94°C for 5 min; (2) 35 cycles of 94°C for 45 s, 55°C for 1 min, and 72°C for 3 min; and (3) 72°C for 20 min. The resulting product was size‐verified on a 1% agarose gel, excised at the expected length (~1500–2000 bp), and purified with a QIAquick Gel Extraction Kit (Qiagen, Hilden, Germany) according to the supplier's instructions. The purified amplicon was Sanger‐sequenced by Macrogen (Seoul, Korea). Forward and reverse reads were aligned and assembled using Geneious Prime v2025.1.3 (Biomatters Ltd., Auckland, New Zealand) to obtain the complete 18S rDNA sequence, which was deposited in GenBank under the accession code PV578870.

### 2.4 Phylogenetic Analyses

To determine the phylogenetic position of strain MHF056, we assembled two independent small‐subunit ribosomal RNA gene (18S rDNA) data sets. The first comprised 17 published apusomonad sequences (2 *Chelonemonas*, 2 *Thecamonas*, 2 *Singekia*, 1 *Karpovia*, 1 *Manchomonas*, 2 *Apusomonas*, 2 *Cavaliersmithia*, 1 *Catacumbia*, 1 *Mylnikovi*a, 1 *Multimonas* and 2 *Podomonas*) from Torruella et al. (2023), the sequence of strain MHF056 itself, six closely related environmental sequences, and three species from Breviatea as an outgroup (27 taxa in total). *Chelonemonas masanensis* was excluded from the phylogenetic analyses due to the published sequence being chimeric (Torruella et al. 2023). The second matrix expanded this set with 94 additional environmental V4 sequences retrieved from the Tara Oceans database, yielding 121 taxa. Both data sets were aligned with MAFFT v7 (Katoh and Standley 2013) using the E‐INS‐I algorithm, manually masked, and retained 1703 bp (27‐taxon) and 1697 bp (121‐taxon) of unambiguously aligned positions.

Maximum‐likelihood trees were inferred with IQ‐TREE v1.6.12 (Nguyen et al. 2015); ModelFinder (−m MFP) selected the TIM2 + F + I + R3 model for both the 27‐ and 121‐taxon alignments, and node support was assessed with 1000 ultrafast bootstrap replicates. Bayesian analyses were performed in MrBayes v3.2.7 (Ronquist et al. 2012) using two independent runs of four chains, with a heating parameter of 0.1. The 27‐taxon matrix was run for 5 × 10^6^ generations, whereas the 121‐taxon matrix was run for 3 × 10^7^ generations, each with a 25% burn‐in. In both cases the average standard deviation of split frequencies after burn‐in fell below 0.01, indicating satisfactory convergence of the chains. All alignments and phylogenetic trees are available on request.

### 2.5 Global Distribution Survey

To assess the global distribution of *Hexatilemonas* gen. n., we queried the Ocean Barcoding Atlas (OBA) web application (Vernette et al. 2021; http://oba.mio.osupytheas.fr/ocean‐atlas). The 18S rDNA sequence of strain MHF056 was searched against the Tara Oceans DADA2 18S‐V4 ASV (eukaryote) database hosted in OBA, and all matching ASVs (> 85% of identity, *n* = 168) were incorporated into a phylogenetic analysis (see above). From this, 94 ASVs that clustered within the *Hexatilemonas* clade with highest bootstrap support (ML: 100%) were then used to map the genus's global distribution across every filter‐size fraction (pico−/nanoplankton, 0.8–5 μm; nanoplankton, 5–20 μm; microplankton, 20–180 μm; and mesoplankton, 180–2000 μm) and all depth categories defined in OBA (SRF = surface layer, DCM = deep‐chlorophyll maximum, MIX = mixed layer, MES = mesopelagic zone). Depth data were subsequently aggregated using Tara Oceans database depth profiles into epipelagic (0–200 m) and mesopelagic (200–1000 m) bins and visualised with the ‘ggplot2’ (Wickham 2011) package in R v4.4.2 (R Core Team 2024). There were no Tara Oceans data available for the mesopelagic zone below or marine sediments.

## 3 Results

### 3.1 Light and Scanning Electron Microscopy

**FIGURE 1:**
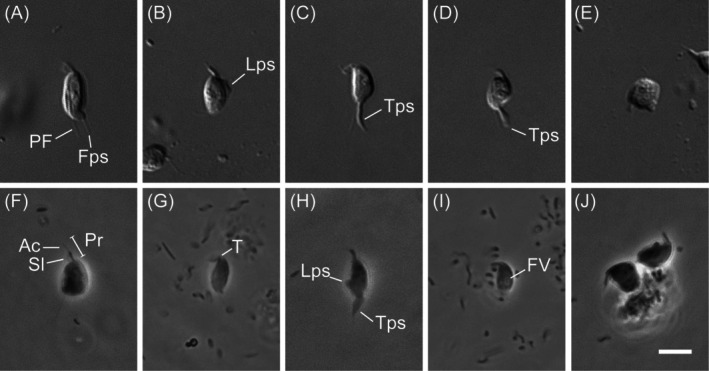
Light micrographs of *Hexatilemonas jangsaensis* gen. et sp. n. (A–E) Differential interference contrast images. (F–J) Phase contrast images. (A–C) display ‘standard’ fusiform ‘*Amastigomonas*’‐type morphology; (D–E) illustrate deformation during directional change. Note composite proboscis (F) consisting of sleeve, acroneme and anterior flagellum. Scale bar = 5 μm for all panels. Ac, acroneme; Fps, filopodia; FV, food vacuole; Lps, lateral pseudopodia; PF, posterior flagellum; Pr, proboscis; Sl, sleeve; T, tusk; Tps, trailing pseudopodia.

The isolate MHF056 exhibited a somewhat plastic main cell body (Figure 1). When it extended into a straight configuration to glide, the cell adopted the fusiform ‘*Amastigomonas*‐type’ morphology typical of most apusomonads (Heiss et al. 2015, 2016; Torruella et al. 2023; Figure 1A–C; Video S1). During directional changes, the cell either bent or contracted its body (Figure 1D,E; Video S1). In its fusiform state, average cell length and width were 5.6 μm (range 5.1–6.4 μm; *n* = 15) and 2.9 μm (2.4–3.8 μm; *n* = 15), respectively. Two flagella were present. The anterior flagellum was enclosed by a sleeve, beyond which an acroneme was visible (Figure 1F); together these elements formed the proboscis, which averaged 2.4 μm in length (range 1.9–2.9 μm; *n* = 15). The proboscis beat laterally to the cell's left and could sweep through angles greater than 90° relative to the cell body (Figure 1G). The posterior flagellum measured 3.3 μm on average (2.7–3.9 μm; *n* = 9) beyond the posterior end of the cell. Although sometimes difficult to observe, it extended as a long trailing strand on the left side, often resembling a filopodium (Figure 1A). Pseudopodia were diverse in form, including trailing filopodia and broad lateral and trailing pseudopodia (Figure 1A–D,H; Video S1). A ‘tusk’ (sensu Heiss et al. 2013) was rarely visible but could occasionally be seen emerging from the right side of the cell body at the base of the proboscis (Figure 1G). Food vacuoles were often identifiable in the cell (e.g., Figure 1I). Cells were frequently observed attached to bacterial aggregates (Figure 1J). Cysts were not observed.

**FIGURE 2:**
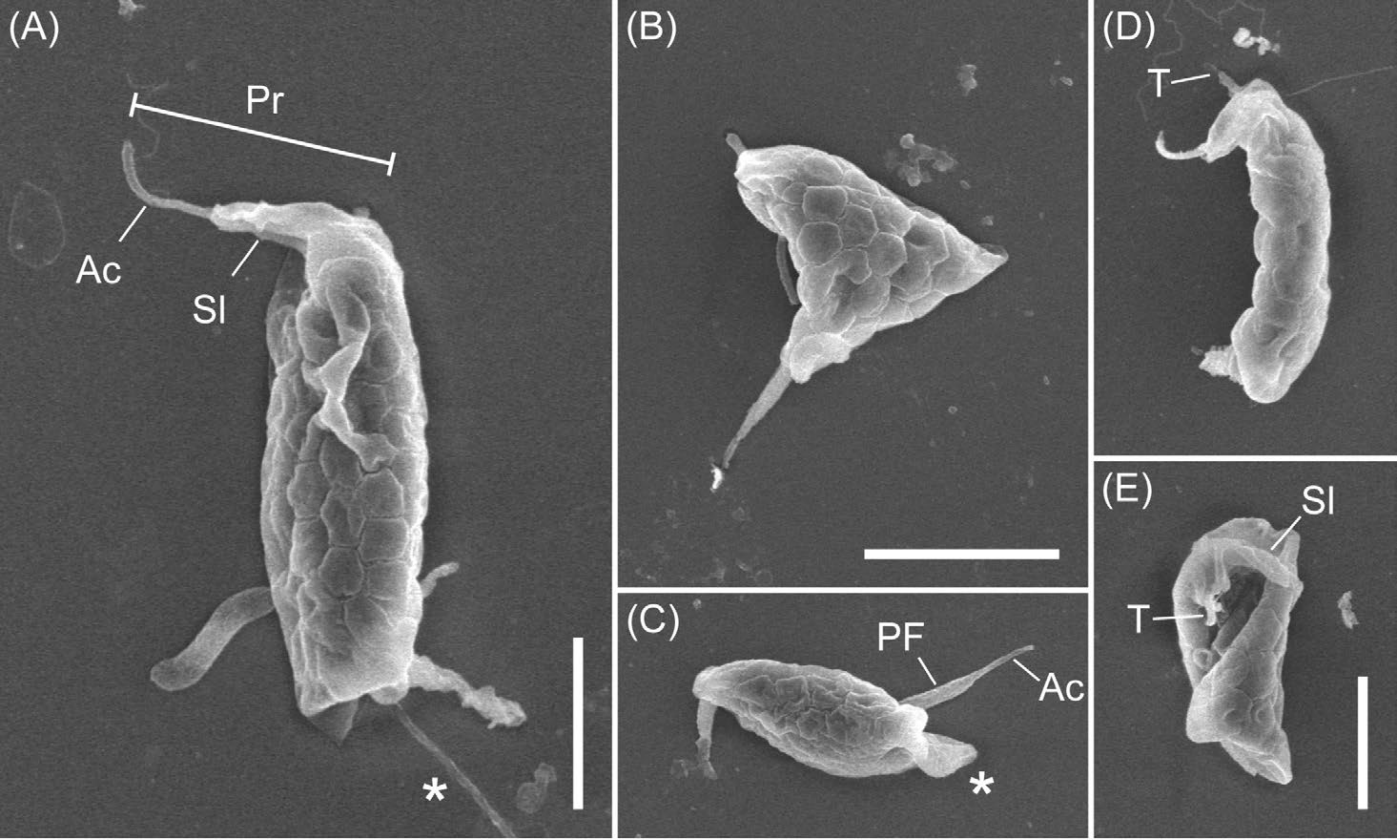
Scanning electron micrographs of *Hexatilemonas jangsaensis* gen. et sp. n. Note hexagonal tile pattern on dorsal surface (A–D) and ‘tusk’ (D, E). Also note filopodium (A), distorted body form (B), and broader trailing pseudopodium alongside posterior flagellum (C). Scale bars = 2.5 μm for panels A, B–C, D–E. Ac, acroneme; Asterisk, pseudopodium; PF, posterior flagellum; Pr, proboscis; Sl, sleeve; T, tusk.

Although the specimens exhibited some shrinkage artefacts, SEM confirmed characteristics previously observed by light microscopy, including the fusiform and distorted cell shapes, the proboscis, the tusk, and the various types of pseudopodia (Figure 2A–E). It also revealed two features that were unobserved under differential interference contrast or phase‐contrast light microscopy. First, it showed an acronematic tip to the posterior flagellum (Figure 2C). Second, the dorsal surface exhibited a distinctive hexagonal, tile‐like pattern (Figure 2). The borders between the tiles appeared to be recessed into the cell.

### 3.2 Feeding Behaviour

**FIGURE 3:**
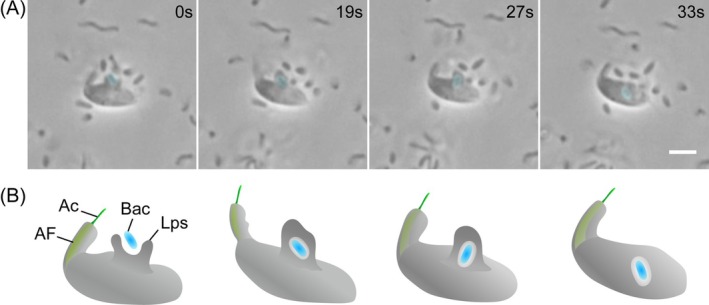
Microscopic images and schematic of the feeding behaviour in *Hexatilemonas jangsaensis* gen. et sp. n. (A) Phase‐contrast micrograph sequence illustrating the ingestion process. (B) Diagrammatic representation of the same feeding cycle. Elapsed time is indicated in upper right panels in (A). Scale bar = 2.5 μm in panel A. Ac, acroneme; AF, anterior flagellum; Bac, bacterium; Blue colour, bacterium; Lps, lateral pseudopodium.

Strain MHF056 captured bacteria with lateral pseudopodia extending from its ventral side, created a phagocytic‐vacuole‐like pocket, and then transported the prey into the cell for digestion (Figure 3; Video S2). All consumed bacteria observed were either aggregated or substrate‐attached (*n* = 5). Microvideo showed this strain to have a sit‐and‐wait predation mode, a type of grasping behaviour: the cell remains stationary and envelops attached bacteria, moving only its proboscis during its feeding behaviour (Figure 3; Video S2).

### 3.3 Sequence Analysis

The 18S rRNA gene sequence obtained from strain MHF056 was 1751 bp long. A BLASTN search in NCBI GenBank showed that its closest match was an environmental sequence, ‘Uncultured Apusozoa clone BS11_E4’ (FN598322), with 98.29% identity. The next‐closest hit was ‘*Thecamonas* sp. 2 Bamfield’ (EU542594), a previously cultured but morphologically undescribed isolate (now dead: Cavalier‐Smith and Chao 2010), with 95.98% identity.

**FIGURE 4:**
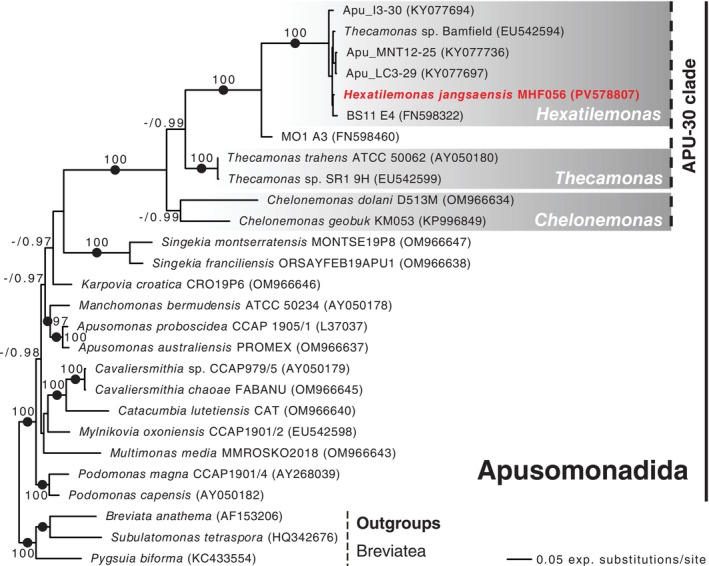
Maximum likelihood phylogenetic tree inferred from small‐subunit (18S) rDNA sequences of 24 apusomonad taxa including *Hexatilemonas* gen. n., with three breviates as outgroup. Ultrafast bootstrap support values from 1000 replicates (> 95%) and Bayesian posterior probability (PP) values are shown along branches. Solid circles indicate PP values of 1 (PP < 0.95 not shown).

**FIGURE 5:**
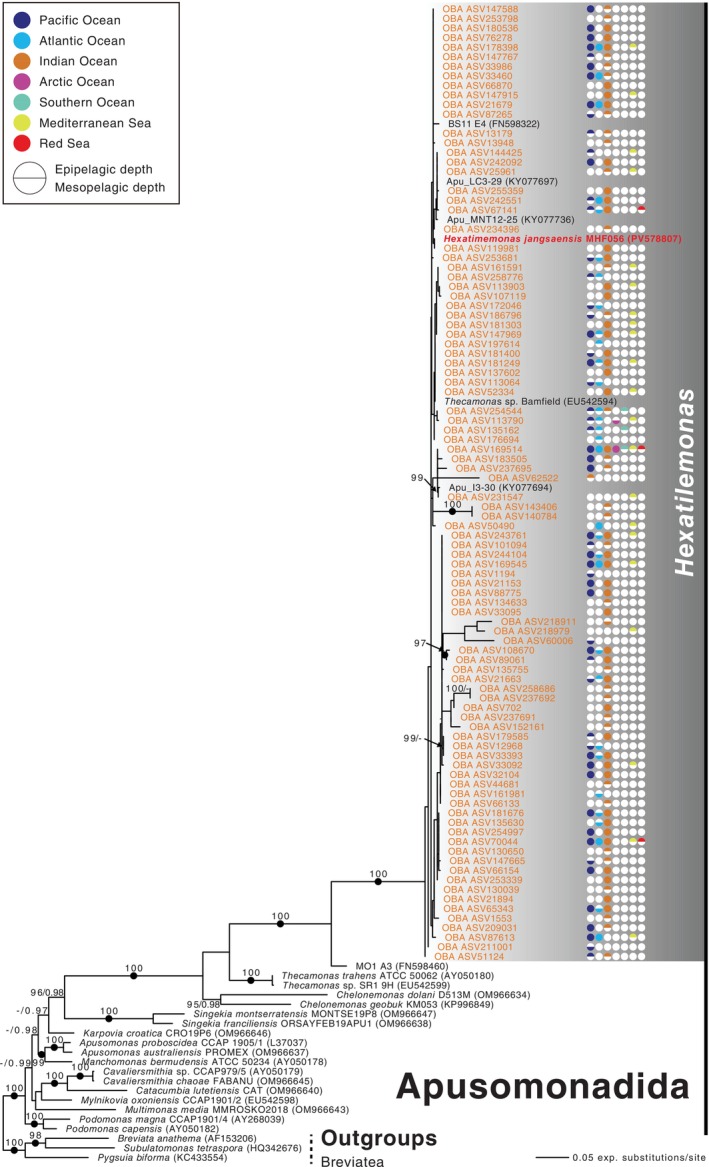
Maximum‐likelihood phylogenetic tree inferred from 94 hypervariable V4 region ASVs matching *Hexatilemonas* gen. n., 24 other apusomonads, and three breviates (as outgroup). Numbers at branches indicate ultrafast bootstrap support values (> 95%) and Bayesian posterior probabilities (PP; PP < 0.95 not shown). Solid circles indicate PP values of 1.

Phylogenetic analyses of the 18S rRNA gene likewise placed isolate MHF056 within a group of the APU‐30 clade that contains only morphologically undescribed isolates and environmental sequences. In a tree based on only full‐length 18S sequences (the 27‐taxon dataset), a clade including MHF056, four environmental samples (Apu_I3‐30, Apu_MNT12‐25, Apu_LC3‐29, and ES11 E4), and ‘*Thecamonas* sp. Bamfield’ was recovered with maximal support from both ultrafast bootstrapping and Bayesian analysis (Figure 4). This clade expanded substantially when environmental sequences from the Tara Oceans database were added (in the 121‐taxon dataset). In this case, the clade also included 94 environmental sequences, again with maximal support (Figure 5).

The arrangement of subclades within the APU‐30 clades was only partially resolved. Both Bayesian and maximum‐likelihood analyses recovered a sister relationship between the subclade containing MHF056 and *Thecamonas* spp., to the exclusion of *Chelonemonas*, and both in the 27‐taxon (Figure 4) and 121‐taxon (Figure 5) datasets. However, while the Bayesian analysis offered near‐maximal (27‐taxon) to maximal (121‐taxon) support for this topology, ultrafast bootstrapping did not support it at all in either dataset. Furthermore, ultrafast bootstrapping failed to support the genus *Chelonemonas* as a whole in the 27‐taxon dataset, and only marginally supported it in the 121‐taxon dataset. Nevertheless, the APU‐30 clade as a whole was recovered with maximal support in all analyses.

### 3.4 Global Distribution Pattern

**FIGURE 6:**
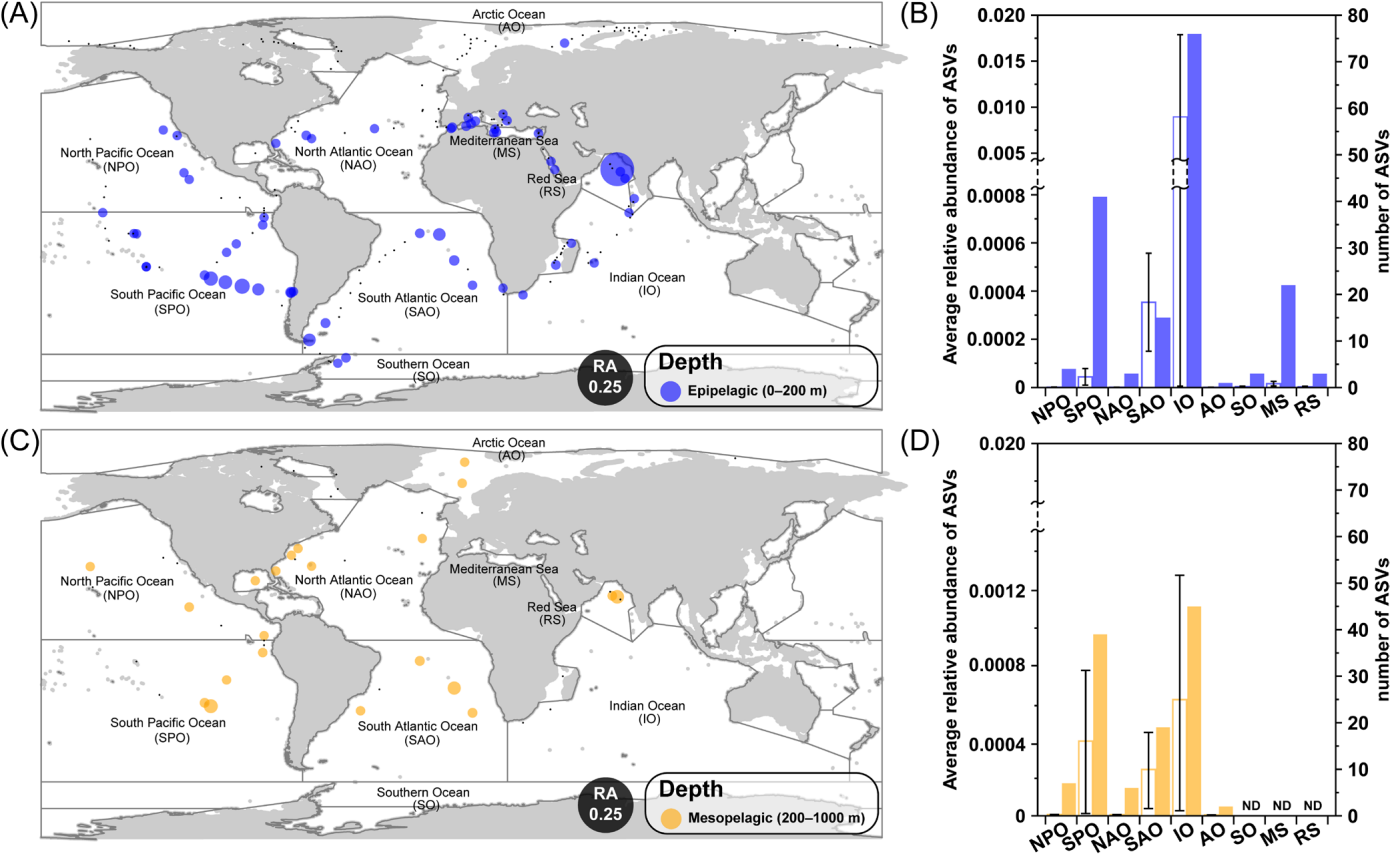
Global distribution pattern maps of *Hexatilemonas* gen. n., showing relative abundance of amplicon sequence variants (ASVs) of the V4 region of 18S rDNA, generated using the Ocean Barcoding Atlas (OBA) web application (http://oba.mio.osupytheas.fr/ocean‐atlas). Panels A and C show epipelagic depth and mesopelagic depth profiles, respectively, of ASVs matching *Hexatilemonas*. Each circle is divided into an upper semicircle and a lower semicircle, representing epipelagic and mesopelagic depths, respectively. Colour indicates ocean regions and the occurrence of *Hexatilemonas*. Small black dots indicate sampled sites with absence of *Hexatilemonas* ASVs. Panels B and D show the average relative abundance (open bars) and the number of ASVs (closed bars). ND, not detected; RA, relative abundance.

We assessed the global distribution of *Hexatilemonas* gen. n. with the Ocean Barcoding Atlas web application. The best‐matching sequences were not restricted to a particular area but were detected widely in both the epipelagic and mesopelagic zones worldwide (Figure 6). Tara environmental sequences affiliated with the genus *Hexatilemonas* occurred in 29.2% (59/202) of all epipelagic samples but in 47.7% (21/44) of all mesopelagic samples. Overall, the Tara Oceans database contained a total of 94 environmental sequences that fell within the *Hexatilemonas* clade, sharing 86.1%–100% sequence identity and corresponding to differences of 0–50 nucleotides out of approximately 370 nucleotides (Figure 5, orange text). The highest relative abundance of these sequences occurred at station 039 in the Indian Ocean, accounting for 0.07 in the epipelagic layer and 0.002 in the mesopelagic layer (Figure 6A,C). The largest numbers and average relative abundance (mean ± SD) of *Hexatilemonas* sequences were detected in the Indian Ocean (epipelagic = 76 ASVs and 0.009 ± 0.009; mesopelagic = 45 ASVs and 0.0006 ± 0.0006), followed by the South Pacific Ocean (epipelagic = 41 ASVs and 0.00004 ± 0.00003; mesopelagic = 39 ASVs and 0.0004 ± 0.0004) and South Atlantic Ocean (epipelagic = 15 ASVs and 0.0004 ± 0.0002; mesopelagic = 19 ASVs and 0.0003 ± 0.0002) (Figure 6B,D).

## 4 Discussion

Light microscopy and scanning electron microscopy observations show that MHF056 shares several hallmark features with *Chelonemonas*: a fusiform ‘*Amastigomonas*’‐type body, a tusk, and an apparent hexagonal segmentation in the dorsal pellicle (Heiss et al. 2015). However, the junctions between polygons in MHF056 pellicle form depressed grooves; in *Chelonemonas*, by contrast, the polygonal junctions are raised. Also, an acroneme on the posterior flagellum is clearly visible in MHF056, but has never been reported in *Chelonemonas*. The stable co‐occurrence of these two traits, along with its phylogenetic position, supports MHF056's placement in a new genus, *Hexatilemonas*.

Our new strain is the first representative in stable culture of a long and diverse branch within the APU‐30 clade. Its placement with maximal support in both ML and Bayesian trees (Figures 4 and 5) confirms that this lineage is phylogenetically distinct from the previously characterised APU‐30 genera *Chelonemonas* and *Thecamonas* (Cavalier‐Smith and Chao 2010; Heiss et al. 2015; Torruella et al. 2017, 2023). Notably, the *Hexatilemonas* clade, along with the closely related MO1 A3 sequence, shows very short internal branches (Figure 4). This pattern remains consistent after the addition of 94 Tara Oceans sequences (Figure 5). Also, this result is consistent with previous phylogenetic studies showing that the *Hexatilemonas* clade was robustly sister to the uncultured apusomonad MO1 A3, which was retrieved from a deep hydrothermal habitat in the Atlantic Ocean (Torruella et al. 2017, 2023). Culturing *Hexatilemonas* therefore closes a critical gap in the systematics of Apusomonadida and provides a living model for future phylogenomic work on one of the two opisthokont sister groups.

Microvideo observations revealed a type of grasping behaviour in *Hexatilemonas jangsaensis*, a behaviour that has also been observed in other apusomonads, specifically in the freshwater *Catacumbia lutetiensis* (Torruella et al. 2023). A similar feeding mode was reported by Artolozaga et al. (2000), who observed grasping ‘*Amastigomonas*’‐like flagellates exclusively inside freshly formed macroaggregate which may be particulate substrates such as marine snow, floating biofilm, or microplastic that host attached prey, but not in the surrounding water column or water interphase. This result highlighted their strong preference for particle‐associated microhabitats. This strategy is likely an efficient way for heterotrophic flagellates to feed preferentially on attached or benthic bacteria (Boenigk and Arndt 2002). However, we note that Kiørboe et al. (2002) showed that motile bacteria rapidly colonise aggregates, whereas non‐motile bacteria do not, suggesting that we not rule out the possibility that motile bacterial species may also be exploited as prey by *Hexatilemonas*, although we did not observe this.

V4 metabarcoding tags show that *Hexatilemonas* occurs in every major ocean basin and at two broadly different depth strata (epi‐ and mesopelagic: Figure 6). Its patchy but global detection pattern is typical of ‘hidden’ microbial guilds that rely on passive transport by currents rather than bloom dynamics. Relative abundances rarely exceeded 0.01 of total V4 reads, placing *Hexatilemonas* among the least abundant heterotrophic flagellates (HFs) documented so far. Such rarity is consistent with earlier reports that apusomonads, together with thaumatomastigids, account for as little as 1% of benthic HF biomass (Arndt et al. 2000) and < 0.1% of pelagic eukaryotic reads (de Vargas et al. 2015). Nevertheless, the genus's widespread occurrence implies efficient dispersal and broad environmental tolerance, an observation that expands the previous finding of APU‐30 members in both coastal and hydrothermal vent microhabitats (Torruella et al. 2017). The relative abundance attributable to *Hexatilemonas* from both epipelagic and mesopelagic layers suggests tolerance to steep gradients in light, temperature (range: −0.8°C–30.6°C) and oxygen (range: 0.8–398.3 μmol/kg). These are attributes that appear common to other, phylogenetically divergent apusomonad lineages (Torruella et al. 2017). The Indian Ocean's high environmental sequence number and average relative abundance of *Hexatilemonas* (epipelagic = 76 ASVs and 0.009 ± 0.009; mesopelagic = 45 ASVs and 0.0006 ± 0.0006) may be the result of dust deposition into it, and subsequent bacterial colonisation on particulate matter, creating resource patches ideally suited to attached bacterivores. Although no direct quantitative study has yet been carried out in the Indian Ocean, case studies from other regions—for example the Arabian Sea (Guieu et al. 2019) and the eastern Mediterranean (Pitta et al. 2017)—show that bacterial assemblages on particle surfaces increase sharply after Saharan or Asian dust deposition (respectively) and that the composition of micro‐grazers shifts accordingly, lending support to the plausibility of this mechanism.

Although *Hexatilemonas* represents only a minor fraction of pelagic microbial biomass, its cosmopolitan presence and particle‐associated lifestyle position it as a potential regulator of aggregated material and the microbes that colonise it. Culturing *H. jangsaensis* now permits direct measurement of ingestion rates, prey specificity, and respiratory quotas under controlled oxygen and pressure conditions, thereby refining oceanic carbon‐flux models that currently ignore apusomonads. Key next steps include metatranscriptomic surveys to verify in situ metabolic activity and isolation of additional strains along latitudinal and depth gradients to test whether the ‘*Hexatilemonas*‐type’ tiled theca and short posterior flagellum are universal ecological adaptations or regional variants. Meanwhile, integrating metabolic and occurrence data with particle‐tracking models will clarify whether *Hexatilemonas* acts chiefly as a prokaryotic grazer, a detritus coloniser, or both, as well as how a once‐‘invisible’ lineage contributes to marine biogeochemical cycling.

## 5 Taxonomy

Eukarya: Amorphea: Obazoa: Apusomonadida Karpov and Myľnikov 1989: Apusomonadidae Karpov and Myľnikov 1989: Thecamonadinae Larsen and Patterson 1990.

### 5.1 *Hexatilemonas* Jeong et al., n. gen.

*Description*: Small (~5.5‐μm) ‘amastigomonad‐type’ cells (sensu Heiss et al. 2015, 2016). Hexagonally segmented dorsal pellicle with recessed boundaries between segments visible under scanning electron microscopy. Proboscis approximately half body length, divided roughly equally into sleeved region with fully developed axoneme and protruding axoneme. Posterior flagellum shorter than cell (~3.3 μm long), acronematic. ‘Tusk’ (sensu Heiss et al. 2013) small (< 0.5 μm long), often inconspicuous. Cytoplasm largely ungranulated. Cell body plastic, especially when changing direction.

*Diagnosis*: Differs from *Thecamonas* in having a segmented pellicle and smaller ‘tusk’; differs from *Chelonemonas* in its pellicle margins being recessed, while *Chelonemonas'* margins are ridge‐like; also differs from *Chelonemonas* in having acronematic posterior flagellum.

*Etymology*: From Latin hexagonum (hexagon) + Neo‐Latin tile (tile) + Greek monas (a single unit, protist), referring to the diagnostic hexagonal‐tile theca and solitary flagellate habit. *Hexatilemonas* is a first‐declension feminine Latin noun.

*Type species*: *Hexatilemonas jangsaensis* Jeong et al., n. sp.

*Other species*: *Hexatilemonas* sp. Bamfield, n. comb. (basionym *Thecamonas* sp. Bamfield Cavalier‐Smith and Chao 2010).

*ZooBank registration*: urn:lsid:zoobank.org:pub:CBCCCF12‐ CC09‐448D‐98E2‐0B90A604C338.

### 5.2 *Hexatilemonas jangsaensis* Jeong et al., n. sp.

*Description*: As for genus *Hexatilemonas* Jeong et al. this work.

*Type strain*: MHF056.

*Isolator*: Dong Hyuk Jeong.

*Type locality*: Surface water–sediment interface, Jangsa Beach, Yeongdeok, Republic of Korea (36° 16′ 48.4″ N, 129° 22′ 40.8″ E).

*Etymology*: The species epithet *jangsaensis* is derived from Jangsa Beach in Yeongdeok, Republic of Korea, the locality where strain MHF056 was isolated.

*Gene sequence*: The full 18S rRNA gene sequence from *Hexatilemonas jangsaensis* (strain MHF056) was deposited in GenBank with accession number PV578807.

*ZooBank registration*: urn:lsid:zoobank.org:pub:C3D022C1‐3E3C‐4148‐BAA0‐2924025D30C8.

## Author Contributions

**Dong Hyuk Jeong:** data curation, formal analysis, investigation, methodology, resources, validation, visualisation, writing – original draft preparation, writing – review and editing. **Hyeon Been Lee:** investigation, writing – review and editing. **Da Yeong Ji:** writing – review and editing. **Aaron A. Heiss:** formal analysis, investigation, writing – review and editing. **Jong Soo Park:** conceptualisation, data curation, funding acquisition, project administration, supervision, writing – review and editing.

## Funding

This work was supported by the Korea Institute of Marine Science and Technology promotion (RS‐2025‐02307311) and the National Research Foundation of Korea (RS‐2022‐NR075591).

## Conflicts of Interest

The authors declare no conflicts of interest.

## Supporting information


**Video S1:** Differential interference contrast and phase contrast video showing gliding movement of *Hexatilemonas jangsaensis* gen. n., sp. n. Scale bar = 5 μm.

**Video S2:** Phase contrast video showing feeding behaviour of *Hexatilemonas jangsaensis* gen. n., sp. n. Scale bar = 5 μm. The rod‐shaped bacterial prey is shown in red.

## Data Availability

The original alignments, masked sites, and phylogenetic trees have been deposited in FigShare: https://doi.org/10.6084/m9.figshare.31408851. All other data generated or analysed during this study are included in this published article.
